# Cytokine changes during treatment of anti-Caspr2 encephalitis: a case report

**DOI:** 10.1186/s12883-020-01879-x

**Published:** 2020-08-13

**Authors:** Yi-Chia Wei, Chia-Lun Wu, Wei-Chieh Weng

**Affiliations:** 1 Department of Neurology, Chang Gung Memorial Hospital, No. 222, Maijin Rd., Anle Dist, Keelung City, 204 Taiwan; 2 Community Medicine Research Center, Chang Gung Memorial, Hospital, Keelung, Taiwan; 3 Collage of Medicine, Chang Gung University, Taoyuan, Taiwan

**Keywords:** Cytokine, Blood, Caspr2, Autoimmune encephalitis, Case report

## Abstract

**Background:**

Cytokines are effective molecules of immune reactions. They work in inflammatory sites as well as circulate in the blood. Cytokines in the cerebrospinal fluid have been suggested to be markers of autoimmune encephalitis and reflect disease progression. However, studies on blood cytokines in autoimmune encephalitis are scarce. We report a case presenting with serial changes in blood cytokine levels in a male patient with anti-contactin-associated protein 2 (Caspr2) encephalitis.

**Case presentation:**

A 61-year-old man without systemic disease presented with ataxia and speech disturbance 1 week. After admission, he further developed visual hallucinations, psychosis, and consciousness deterioration. Brain magnetic resonance imaging and infection and tumor surveillances were negative. 18F-fluorodeoxyglucose positron emission tomography of brain revealed frontal and occipital hypometabolism and anterior cingulate gyrus and mesial temporal hypermetabolism. Autoimmune studies confirmed Caspr2 antibodies in his blood. After receiving a diagnosis of anti-Caspr2 encephalitis, the patient received steroids, plasmapheresis, and zonisamide. He recovered well and was totally independent 6 months after disease onset.

A cytokine profiler array kit was used to investigate neuroimmune mechanisms during the disease course. Several cytokines showed significant changes in plasma levels, such as B cell activating factor for B cell proliferation; thymus and activation-regulated chemokine for T cell chemoattraction; soluble CD40 ligand for Th2 cell mediation; C5/C5a for complement activation; brain-derived neurotrophic factor for neuronal survival response; and dipeptidyl peptidase 4, retinol binding protein, dickkopf-related protein, and epidermal growth factor for response to environmental provocation. The concentration of cytokines was verified using Luminex multiplexing assay.

**Conclusions:**

Due to their easy accessibility, blood cytokines are potential biomarkers of autoimmune encephalitis. Based on the investigating platform of this single case study, future larger scale studies are warranted.

## Background

The pathogenesis of surface-antigen-associated autoimmune encephalitis involves multiple immune mechanisms. Autopsies or biopsies were rarely performed in patients with autoimmune encephalitis; however, pathological studies have noted the following characteristics: immunoglobin depositions on the brain parenchyma, CD3-positive T lymphocyte infiltration in the cerebral cortex, CD20-positive B lymphocyte cuffing in the perivascular area, and CD68-positive macrophage and microglia infiltration. Another unique observation is the presence of CD8 cytotoxic T cells in approximately half of the cases with surface-antigen-associated encephalitis [1–3]. However, disease-specific variations, such as complement activation in anti- contactin-associated protein 2 (Caspr2) encephalitis but not in other types of surface-antigen-associated autoimmune encephalitis, cannot be neglected. In addition, individual-specific variations affect immune response to autoimmune encephalitis, for example, the degree of inflammatory cell infiltration varied considerably in different patients with anti-Caspr2 encephalitis [1].

Cytokines, the reactive molecules of immune reactions, reflect immune mechanisms and are therefore potential biomarkers of immune-mediated diseases. Although cerebrospinal fluid (CSF) biomarkers have been studied in detail for their role in autoimmune encephalitis [4, 5], blood-based biomarkers are scarcely discussed. Therefore, we examined the blood cytokine profiles of a patient with autoimmune encephalitis associated with anti-Caspr2 antibodies and aimed to identify potential blood-based biomarkers.

## Case presentation

### Clinical scenario

A 61-year-old man with no systemic disease developed acute onset of slurred speech and drooling, with subsequent wide-based gait, ataxia of upper limbs, and slurred and scanning speech for 1 week. After a week of being admitted to our Neurology ward, he developed psychosis, insomnia, agitation, delusion of persecution, vivid visual hallucinations of colorful stereoscopic images, and then confusion and drowsiness. Upon reviewing his recent exposure and contact history, we discovered that he had inspected a long-closed underground construction site 2 days before symptom onset. However, he showed no sign of fever or upper airway symptoms.

**Fig. 1:**
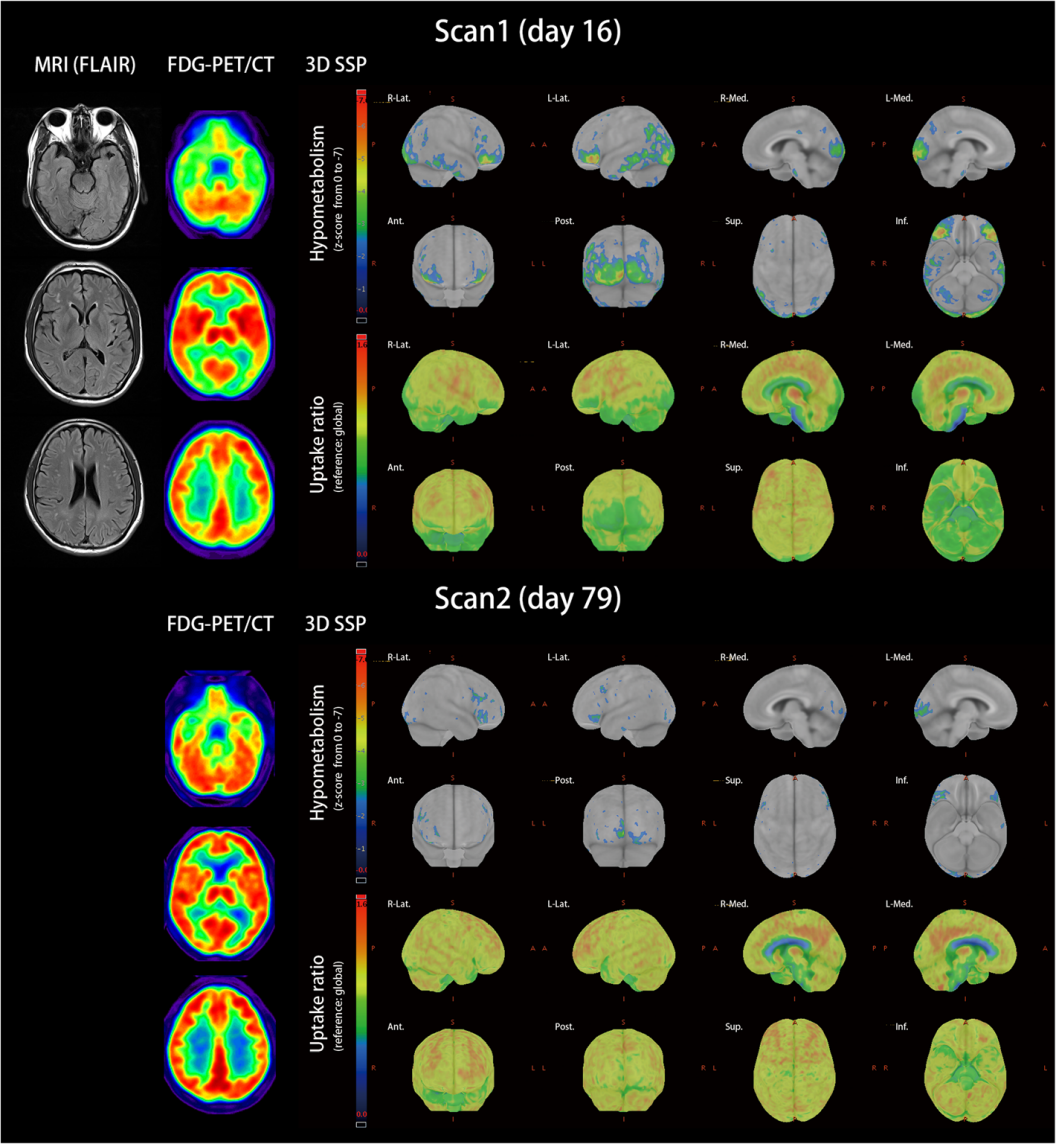
Brain image of MRI and FDG-PET/CT. Initial brain MRI was nonspecific. Fluid-attenuated inversion recovery (FLAIR) imaging did not reveal hyperintensity. FDG-PET/CT was processed through stereo and quantitative analysis by using the standardized z-score with the reference of global brain metabolism. Glucose metabolism was visualized using three-dimensional stereotactic surface projection (3D-SSP). In Scan1 on day 16, the brain exhibited hypometabolism in the medial frontal, orbitofrontal, and occipital lobes and hypermetabolism in the anterior cingulate gyrus and mesial temporal areas. In the first scan, the patient was confused and disoriented. The follow-up scan on day 79 showed resolution of focal hypo- and hypermetabolism. During the second scan, the patient was clear in consciousness, with remaining scanning speech, mild ataxic gait, and some difficulties in calculation

**Fig. 2:**
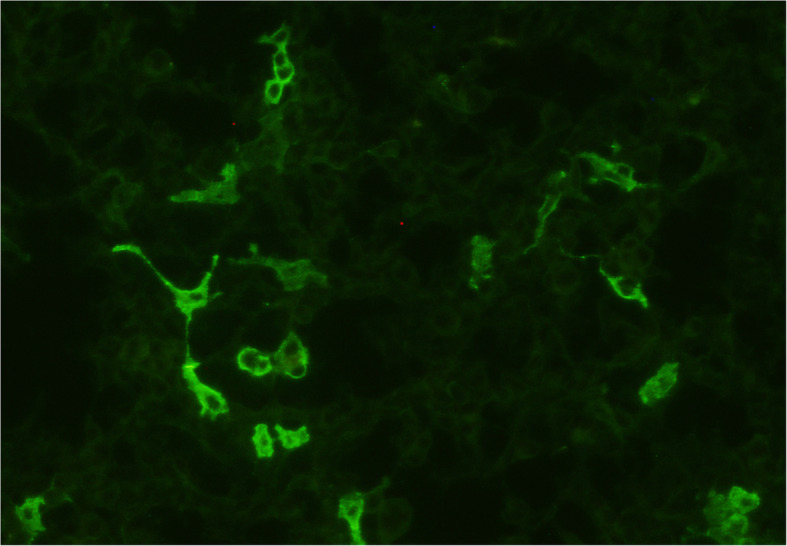
Immunofluorescence of anti-Caspr2 antibodies in the patient’s plasma. The anti-Caspr2 antibodies bound on the Caspr2 antigen expressed by the HEK293 cells and visualized by the immunofluorescence of fluorescein on the second antibody (EUROIMMUN, IIFT: Autoimmune Encephalitis Mosaic 6, Germany)

The initial magnetic resonance imaging (MRI) of the brain was normal (Fig. 1). A WBC level of 1/μL, protein level of 37 mg/dL, and IgG index of 0.69 were noted in CSF studies. Infectious and metabolic surveillances were unremarkable. Anti-Caspr2 antibodies were detected in his blood by using a cell-based indirect immunofluorescence test of autoimmune encephalitis (EUROIMMUN, Germany) (Fig. 2). Electromyography was essentially normal, with no neuromyotonic changes in peripheral nerves. Tests for malignancy, including whole-body 18F-fluorodeoxyglucose positron emission tomography computed tomography (FDG-PET/CT) and tumor marker tests, were negative.

Serial functional studies revealed diffuse cortical dysfunctions. Brain FDG-PET/CT revealed prefrontal and occipital hypometabolism and mesial temporal hypermetabolism, which resolved in the follow-up scan 2 months later (Fig. 1). Intermittent diffuse theta waves were recorded on scalp electroencephalogram. Visual evoked potential revealed bilateral prolonged P100 latencies with poor waveforms. Bilateral concentric peripheral visual field loss was detected on visual field examination. The retinography was normal.

Under the diagnosis of anti-Caspr2 encephalitis, the patient was started on methylprednisolone pulse therapy (1 g/day) for 5 days, and continued with oral prednisolone therapy (1 mg/kg/day) and 10 sessions of plasmapheresis. A voltage-gated sodium channel inhibitor, Zonisamide (200 mg/day), was used because blocking sodium channels was beneficial in controlling neuronal hyperexcitability of voltage-gated potassium channel antibody-associated disorders [6]. The patient recovered well. He was completely independent (modified Rankin Scale 1) six months after disease onset with only mild scanning speech.

### Cytokine profiler array

By using protein blotting membrane-based multiplexing immunoassay (Proteome Profiler Array, Human XL Cytokine Array Kit, R&D Systems, Minneapolis, USA), we examined the profiles of 105 cytokines. The table in Supplementary 1 presents the complete list of tested cytokines. Three time points of plasma sampling were before immunotherapy (day 10 from symptom onset), during immunotherapy (day 28, after steroid pulse therapy and five sessions of plasmapheresis), and after immunotherapy (day 42, after another five sessions of plasmapheresis). The mechanism of the cytokine array was immunoblotting with a cellular membrane coated with antibodies specific to each cytokine. To be detected in the cytokine array, the analytes in blood samples bound onto the matched primary antibodies, and then worked with second antibodies and visualized after reacting with the substrates. Intensities of immunoblotting represented cytokine levels in each blood sample. The relative intensities of cytokine were quantified by the ratio of intensity of each cytokine to the average intensity of three internal references.

**Fig. 3:**
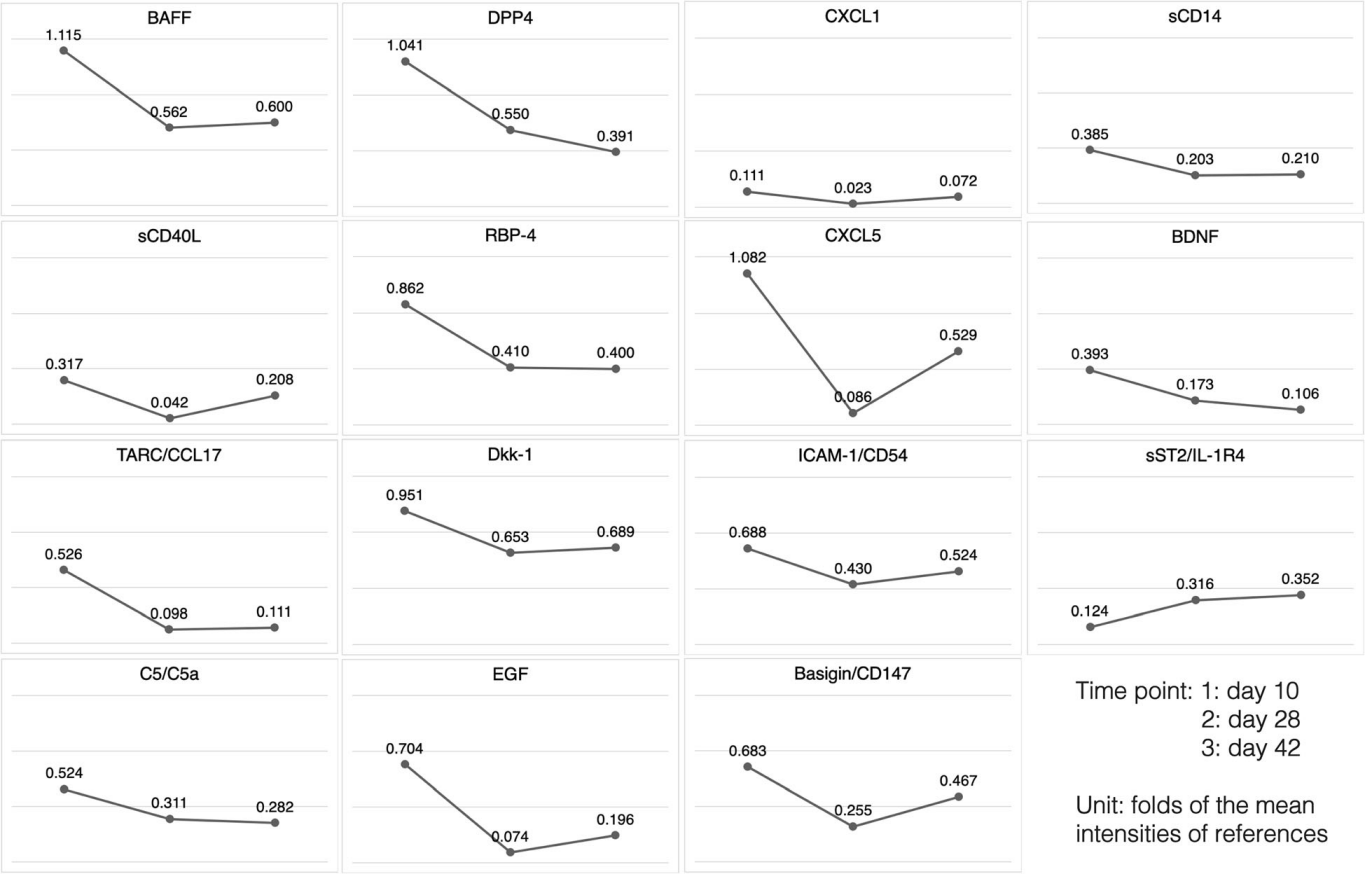
The cytokine profiler array was used to measure relative intensities of cytokines (Proteome Profiler Array, Human XL Cytokine Array Kit, R&D Systems) at three time points: day 10 (before immunotherapy), day 28 (after steroid pulse therapy and 5 sessions of plasmapheresis), and day 42 (after the other 5 sessions of plasmapheresis). The relative intensity was determined by the ratio of intensity of each cytokine to the average intensity of three internal references. For example, the BAFF intensity was 1.115-fold of the mean intensities of references at day 10. The dividing lines in each unit figure represented 0.4-, 0.8-, 1.2-fold intensities to references

A significant decline in relative intensities in serial plasma samples was found for B cell activating factor (BAFF) (1.115-, 0.562-, and 0.600-folds to reference on day 10, 28, and 42, respectively), soluble CD40 ligand (sCD40L) (0.317-, 0.042-, and 0.208-folds), thymus and activation-regulated chemokine (TARC/CCL17) (0.526-, 0.098-, and 0.111-folds), and complement active components C5/C5a (0.524-, 0.311-, and 0.282-folds). The initial increment was observed in brain-derived neurotrophic factor (BDNF) (0.393-, 0.173-, and 0.106-folds), a neuronal survival and neuroplasticity marker. Early in the disease onset, we also observed an increase in the cytokines of innate immunity activation, including dipeptidyl peptidase 4 (DPP4), retinol binding protein (RBP-4), dickkopf-related protein (Dkk-1), epidermal growth factor (EGF), chemokine (C-X-C motif) ligand 1 (CXCL1), chemokine (C-X-C motif) ligand 5 (CXCL5), intercellular adhesion molecule 1 (ICAM-1/CD54), Basigin/CD147, and soluble CD14 (sCD14). Different from the other cytokines, soluble ST2 receptor (sST2/IL1-R4) increased during treatment (0.124-, 0.316-, and 0.352-folds), indicating the activation of anti-inflammatory responses [7] (Fig. 3 and Supplementary 2).

### Verification by using Luminex multiplexing assay

We selected the following five cytokines with representative roles of immune mechanisms and with significant changes by a fold change of > 50% from baseline for verification: BAFF, sCD40L, TARC/CCL17, C5/C5a, and BDNF. The fluorescent bead-based multiplexing immunoassay (Magnetic Luminex Assay: Human Premixed Multi-Analyte Kit, R&D Systems, Minneapolis, USA) was run on Luminex 200, a dual-laser, flow-based sorting and detection platform [8, 9]. Magnetic microparticles precoated with cytokine-specific capture antibodies and embedded with fluorophores were reacted with plasma samples. Next, a cocktail of biotinylated detection antibodies specific to the cytokines were bound to the cytokine-capture-antibody-microparticle complexes. The Streptavidin-phycoerythrin conjugate was then bound to the detection antibodies and excited by lasers in the Luminex analyzer to determine the concentration of each cytokine.

**Fig. 4:**
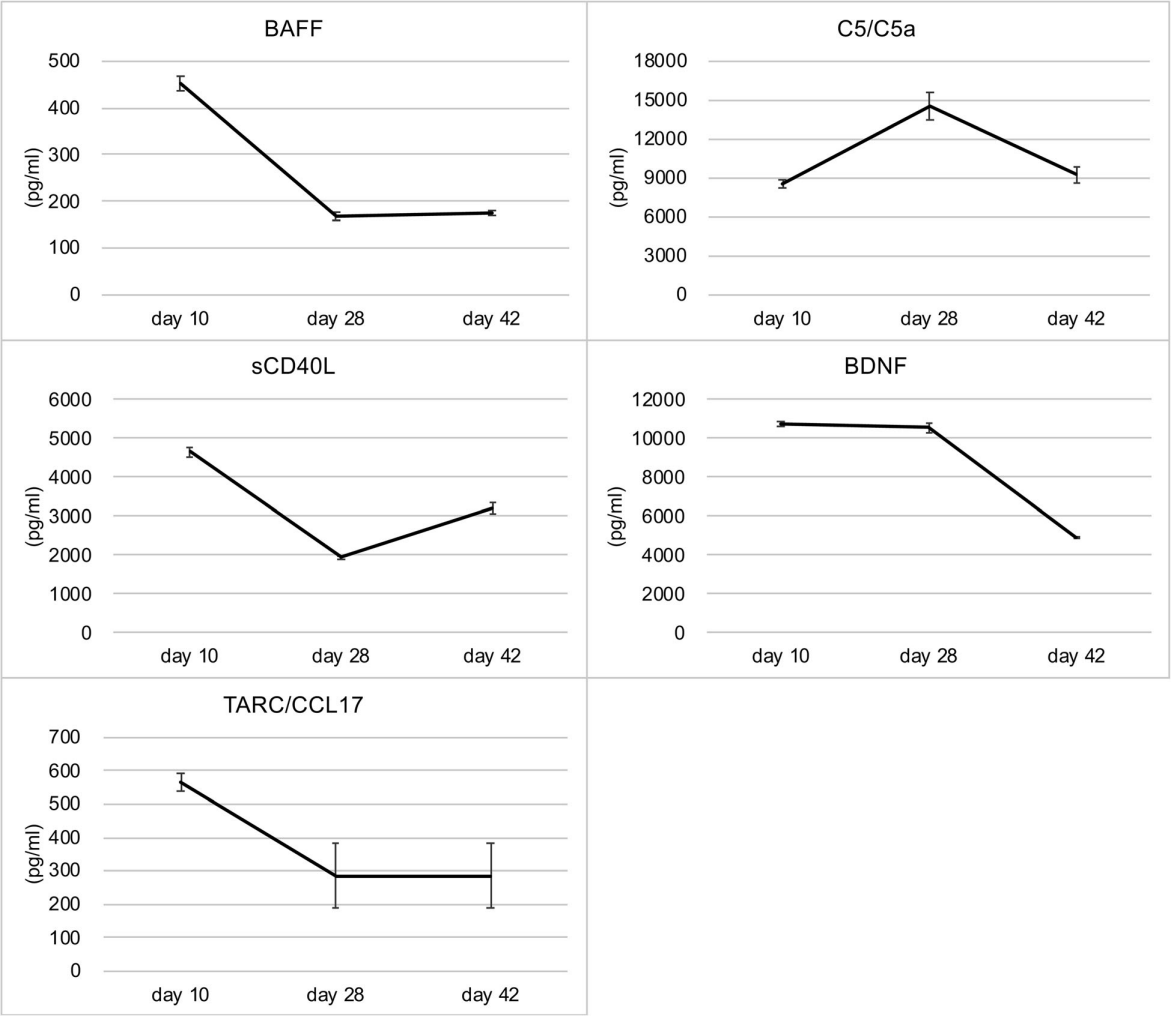
Verification of plasma cytokine changes by using the Luminex multiplexing assay (Magnetic Luminex Assay: Human Premixed Multi-Analyte Kit, R&D Systems, Minneapolis, USA). Concentration of BAFF, TARC/CCL17, sCD40L, C5/C5a, and BDNF in the same plasma samples as used in cytokine profiler array were quantified using the Luminex assay under three repeated tests. Unit of cytokine concentration was pg/mL

In serial plasma samples from the patient, the concentrations of BAFF (453.3 ± 15.4 pg/mL on day 10, 167.7 ± 9.7 pg/mL on day 28, 175.7 ± 4.6 on day 42), sCD40L (4647.2 ± 115.2, 1945.7 ± 48.9, 3189.6 ± 144.5 pg/mL), and TARC/CCL17 (567.1 ± 27.6, 286.0 ± 97.1, 286.0 ± 97.1 pg/mL; Fig. 4 and Supplementary 3) fit the fold changes of immunoblotting intensities in the cytokine array (Fig. 3). Regarding BDNF, the concentration measured using Luminex assay (10,755.3 ± 140.6, 10,543.9 ± 261.3, 4915.8 ± 57.8 pg/mL) and intensities measured using the cytokine array exhibited the same trend of an initial increase and decrease after treatment. Serial-level changes in C5/C5a were partially comparable between concentrations measured using Luminex assay (8577.1 ± 342.2, 14,554.5 ± 1056.3, 9263.0 ± 586.0 pg/mL) and protein intensities measured using the cytokine array.

## Discussion and conclusions

### The case and plasma cytokine changes

**Fig. 5:**
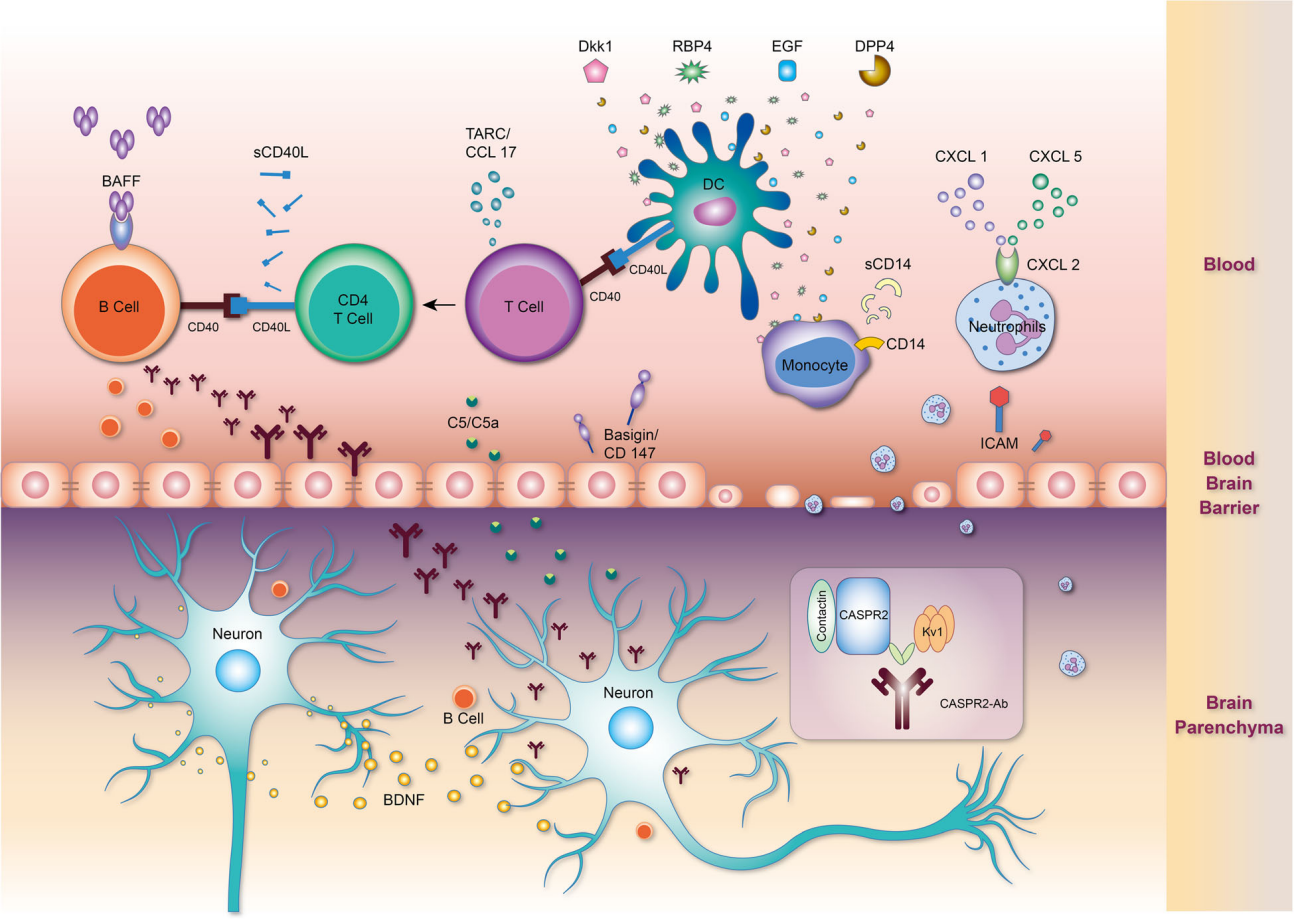
Illustration of immune mechanisms in our case of anti-Caspr2 encephalitis. The cytokines with significant changes in cytokine profiler array (fold change > 50% of the relative intensity) are marked in the illustration. BAFF is a B-cell-stimulating factor. TARC/CCL17 is a T-cell chemokine. sCD40L is an intercellular ligand of Th2 cells to B cells or monocytes. C5/C5a denote complement active components. Dkk-1, DPP4, RBP-5, and EGF are cytokines that respond to environmental stimulation. CXCL1, CXCL5, ICAM-1/CD54, Basigin/CD147, and sCD14 are also cytokines of innate immunity. BDNF is a neuronal survival and neuroplasticity marker secreted by damaged neurons. (This illustration is an original artwork)

We reported a case of anti-Caspr2 encephalitis. Our patient was a 61-year-old man presenting with acute onset progressive cerebellar ataxia, visual hallucination, and consciousness disturbance. We were prompt with his diagnosis and treatment. The patient recovered well after immunotherapy. Studies examining the immune mechanism by using the immunoblotting cytokine array and Luminex multiplexing immunoassay for verification have revealed the involvement of B (BAFF) and T cells (TARC/CCL17), interactions between T and B cells (sCD40L), complement activation (C5/C5a), and neuronal survival reaction (BDNF). A possible provoking factor in our case could be his recent visit to a long-closed underground construction site because his symptoms started shortly after that. Probably, the site was teeming with abundant potential pathogens and environmental stimulating factors. The trigger effects might be reflected in the initially high levels of Dkk-1, RBP-4, EGF, DPP4, and other cytokines of innate immunity (Fig. 5).

BAFF is an important factor for B cell survival and maturation. Increased BAFF levels were observed in blood and inflammatory sites of patients with autoimmune diseases [10]. Antagonism of BAFF is a therapeutic target of autoimmune diseases [11]. A significantly high level of BAFF was reported in the acute stage, which reduced in the recovery stage in the CSF of patients with anti- N-methyl-D-aspartate receptor (NMDAR) encephalitis [5].

TARC/CCL17 is a member of the CC chemokine family. It induces chemotaxis of T cells via CC chemokine receptor 4 that is selectively attracted to Th2 cells in particular [12]. The serum level of TARC/CCL17 is closely related to disease activities of allergic immunity, including atopic dermatitis [13] and bronchial asthma [14].

CD40 receptor is a costimulatory molecule expressed by B cells and antigen-presenting cells. T cells and other nonimmune cells express CD40L, the ligand of CD40. CD40–CD40L binding involves a broad range of immune reactions, promoting both humoral and cellular immunity [15]. The binding reaction also gives rise to sCD40L, the soluble form of CD40L. Serum sCD40L was significantly higher in patients with systemic autoimmune diseases than in controls [16]. Serum sCD40L was relatively low in treated than in untreated patients with autoimmune thrombocytopenia purpura [17]. Thus, CD40L is a therapeutic target on account of its significance in autoimmunity. Several monoclonal antibodies against CD40L are under development [18].

C5/C5a is an active component of the complementary system, which belongs to the innate immune system. Dysregulation of the complement system results in tissue damage in certain autoimmune diseases, including systemic lupus erythematosus (SLE), rheumatoid arthritis, Sjögren’s syndrome, and vasculitis [19]. Patients with anti-Caspr2 encephalitis [20], anti-LGI1 encephalitis [21] and antibody specificity unknown anti-voltage-gated potassium channel encephalitis [1] have exhibited complement deposition in the brain parenchyma. Complement activation seems to be specific for certain types of autoimmune encephalitis and absent in others. For example, complement deposition is not observed in anti-NMDAR encephalitis [22].

BDNF is one of the best studied neurotrophins. Neurodegenerative diseases, such as Alzheimer’s disease and Parkinson’s disease, are characterized by a decrease in BDNF. Therefore, achieving BDNF augmentation has become the goal in treating neurodegenerative diseases [23]. Conversely, inflammation and damage of neurons induce BDNF release. An experimental autoimmune encephalomyelitis mouse model demonstrated the axonal protection function of BDNF [24]. BDNF also played a neuroprotective role in patients with multiple sclerosis [25]. Its level was observed to increase in an animal model in response to the immunomodulatory drug glatiramer acetate [26].

### Cytokines as biomarkers in autoimmune encephalitis

Being both circulating molecules and on-site effective molecules, cytokines are potential biomarkers of autoimmune diseases [27]. They reflect both disease involvement and activity of systemic autoimmune diseases. One study reported that CSF IL-6 and IL-8 were associated with CNS involvement of SLE [28]; another research demonstrated that serum levels of IL-6 and IL-10 were correlated with disease activity of SLE [29].

Although autoimmune encephalitis is less prevalent than systemic autoimmune diseases such as SLE, certain cytokines in the CSF have been identified to reflect intrathecal immune activation in autoimmune encephalitis. For example, the CSF of patients with anti-NMDAR encephalitis displayed higher levels of the B cell survival and proliferative factors BAFF and a proliferation-inducing ligand APRIL than controls [5]. CXCL10 (a chemoattractant of T cell and monocyte/macrophage) and CXCL13 (a B cell chemoattractant) in the CSF were associated with early progression and overall complications of anti-NMDAR encephalitis [30]. CSF CXCL13 levels increase early in disease onset and correlate with treatment response and relapse; therefore, it is considered a biomarker of anti-NMDAR encephalitis [4].

In the literature, whether blood cytokine could detect immune changes in autoimmune encephalitis is unclear. However, circulating cytokines reflect features of disease pathogenesis in other autoimmune neurologic disorders. For example, in B-cell-mediated autoimmunity, higher serum BAFF levels distinguished myasthenia gravis from multiple sclerosis and other nonimmune-mediated neurologic disorders [31]. In another research serum concentration of BAFF was observed to be proportional to the concentration of anti-acetylcholine receptor antibodies [32]. Thus, cytokines in the blood are also potential biomarkers of autoimmune encephalitis. The platform of biomarker identification combined screening and quantitative assays in our report. Future investigations are warranted to confirm the role of BAFF, TARC/CCL17, sCD40L, C5/C5a, and BDNF in anti-Caspr2 encephalitis and other types of autoimmune encephalitis.

However, the factors that may interfere and modify cytokine expression should be cautiously considered in interpreting cytokine biomarkers. Interindividual variability in cytokine levels comes from genetic-mediated response to stimulants [33], age [34], gender [35], and gut microbiota [36]. The expression level of cytokines may also be affected by environmental factors such as seasons [35]. Treatment given to the patient(s), especially immune-modifying drugs, are also potential factors altering blood cytokines. Therefore, this information is required when checking blood cytokine levels in patients with autoimmune encephalitis.

### Method considerations of profiling cytokine levels in disease course

In this case report, we used a commercial protein blotting membrane-based multiplexing immunoassay kit (cytokine array, R&D) to screen cytokines with significant changes during treatment and verified the changes using the fluorescent bead-based multiplexing immunoassay (Luminex assay, R&D). The cytokine array has been applied to identify immune status modifications by comparing diseased people with healthy controls [37], to identify chronic disease in different clinical stages [38], and to compare changes before and after treatment [39].

Although the cytokine array screens several cytokines in a single test, verification using a standard quantification method is necessary. Enzyme-linked immunosorbent assays (ELISA) for individual proteins or bead-based multiplexing immunoassay for multiple analytes could be used as the verification tests. ELISA is the classical method for analyzing cytokine levels. However, it is not practical because of the requirement of sample volumes for multiple assays in measuring multiple analytes. Fluorescent bead-based technology, such as the multiplex assay Luminex, measures multiple analytes in a single sample and facilitates the analysis of cytokine expression patterns for characterizing the immune status. Moreover, it exhibits favorable accuracy in reflecting actual cytokine levels when tested with standardized controls [40]. Excellent correlations between the Luminex assay and ELISA were obtained for the majority of tested cytokines [41]. Serial measurements of relative concentrations of soluble cytokines were demonstrated to reflect the continuous changes in immune response [8]. Therefore, we chose the Luminex assay as the verification tool for cytokine levels in our study.

As observed in our results, not all the curve changes were compatible between the cytokine array and Luminex assay, for example, C5/C5a and BDNF. The membrane-based cytokine array follows the principle of Western blotting and yields semiquantitative results. Because the Luminex assay is a quantitative method, certain discrepancies might exist between semiquantitative and quantitative analyses. We preferred to consider the results of the Luminex for interpretation because they were theoretically more precise than those of the cytokine array. Increasing the number of patients is a way to confirm the reproducibility of these cytokine changes during anti-Caspr2 encephalitis to overcome bias.

### Limitations of the study

Individual variation in cytokine levels is a significant consideration when studying the immune reactions of patients with autoimmune encephalitis [1]. Although several plasma cytokines showed significant changes in this case report, an analysis with a large number of cases is needed to verify our findings. Second, the symptoms observed in autoimmune encephalitis result from intrathecal damages. The accessibility of blood samples is better than that of CSF samples; however, blood cytokines could only indirectly infer the ongoing immune reaction in brain. In other words, blood cytokines reflected systemic immune involvement and indicated that the immune response of autoimmune encephalitis is not restricted to CNS immunity with focal intrathecal inflammation but is also a systemic reaction. Therefore, both pros and cons were observed in interpreting blood cytokine changes in autoimmune encephalitis.

### Conclusions

The cytokine profiler array was used to examine serial blood cytokine changes during the treatment of our patient. The Luminex multiplexing assay was used to verify cytokine changes. The suggested immune mechanisms of pathogenesis were innate immunity activation possibly responding to environment provocation, B cell (BAFF) and T cell (TARC/CCL17 and sCD40L) participation, complement activation (C5/C5a), and neuronal survival response (BDNF). Further investigations should develop blood biomarkers based on this platform.

## Supplementary information

**Additional file 1: Supplementary 1.** Cytokines tested in the cytokine array. **Supplementary 2.** Serial cytokine arrays of the patient’s plasma. **Supplementary 3.** Cytokine concentration determined by Luminex Assay

**Additional file 2: Supplementary 4.** CARE checklist

## Data Availability

The datasets used and/or analyzed during the current study are available from the corresponding author on reasonable request.

## Abbreviations

BAFF: B cell activating factor
BDNF: Brain-derived neurotrophic factor
Caspr2: Contactin-associated protein 2
CXCL1: Chemokine (C-X-C motif) ligand 1
CXCL5: Chemokine (C-X-C motif) ligand 5
Dkk-1: Dickkopf-related protein
DPP4: Dipeptidyl peptidase 4
EGF: Epidermal growth factor
ELISA: Enzyme-linked immunosorbent assays
3D-SSP: Three-dimensional stereotactic surface projection
FDG-PET/CT: ^18^F-Fluorodeoxyglucose positron emission tomography and computed tomography
FLAIR: Fluid-attenuated inversion recovery
ICAM-1/CD54: Intercellular adhesion molecule 1
NMDAR: N-methyl-D-aspartate receptor
RBP-4: Retinol binding protein
sCD14: Soluble CD14
sCD40L: Soluble CD40 ligand
sST2/IL1-R4: Soluble ST2 receptor
SLE: Systemic lupus erythematosus
TARC/CCL17: Thymus and activation-regulated chemokine
