# Supplementary material for: Cytokine changes during treatment of anti-Caspr2 encephalitis: a case report
Source: BMC Neurol. 2020 Aug 13;20:299. doi: 10.1186/s12883-020-01879-x (PMC7425608; doi:10.1186/s12883-020-01879-x)
Supplement: Supplementary file 1 — Additional file 1: Supplementary 1. Cytokines tested in the cytokine array. Supplementary 2. Serial cytokine arrays of the patient’s plasma. Supplementary 3. Cytokine concentration determined by Luminex Assay [file 12883_2020_1879_MOESM1_ESM.docx]

**Supplementary 1. Cytokines tested in the cytokine array**

|  | 1,2 | 3,4 | 5,6 | 7,8 | 9,10 | 11,12 | 13,14 | 15,16 | 17,18 | 19,20 | 21,22 | 23,24 |
| --- | --- | --- | --- | --- | --- | --- | --- | --- | --- | --- | --- | --- |
| A | Reference | Adiponectin | Apolipoprotein A-I | Angiogenin | Angiopoietin-1 | Angiopoietin-2 | BAFF | BDNF | Complement C5/C5a | CD14 | CD30 | Reference |
| B |  | CD40L/ CD154 | Chitinase 3-like 1 | Complement Factor D | C-Reactive Protein | Cripto-1 | Cystatin C | Dkk-1 | DPP4 | EGF | Emmprin |  |
| C |  | CXCL5 | Endoglin/ CD105 | Fas Ligand | FGF basic | FGF-7 | FGF-19 | Flt-3 Ligand | G-CSF | GDF-15/ MIC1 | GM-CSF |  |
| D | CXCL1 | Somatotropin | HGF | ICAM-1/ CD54 | IFN-γ | IGFBP-2 | IGFBP-3 | IL-1α | IL-1β | IL-1ra | IL-2 | IL-3 |
| E | IL-4 | IL-5 | IL-6 | IL-8 | IL-10 | IL-11 | IL-12 p70 | IL-13 | IL-15 | IL-16 | IL-17A | IL-18 Bpa |
| F | IL-19 | IL-22 | IL-23 | IL-24 | IL-27 | IL-31 | IL-32 | IL-33 | IL-34 | CXCL10 | CXCL11 | Kallikrein 3/ PSA |
| G | Leptin | LIF | Lipocalin-2 | MCP-1/ CCL2 | MCP-3/ CCL7 | M-CSF | MIF | MIG/ CXCL9 | MIP-1α/ MIP-1β | MIP-3α | MIP-3β | MMP-9 |
| H | Myeloperoxidase | Osteopontin | PDGF-AA | PDGF-AB/BB | Pentraxin 3 | PF4/ CXCL4 | RAGE | RANTES | RBP-4 | Relaxin-2 | Resistin | SDF-1α/ CXCL12 |
| I | Serpin E1 | SHBG | ST2/ IL1R4 | TARC/ CCL17 | TFF3 | TfR/ CD71 | TGF-α | Thrombospondin-1 | TNF-α | uPAR | VEGF |  |
| J | Reference |  | Vitamin D BP | CD31 | TIM-3 | VCAM-1/CD106 |  |  |  |  |  | Negative Controls |

**Supplementary 2. Serial cytokine arrays of the patient’s plasma**

| Template | 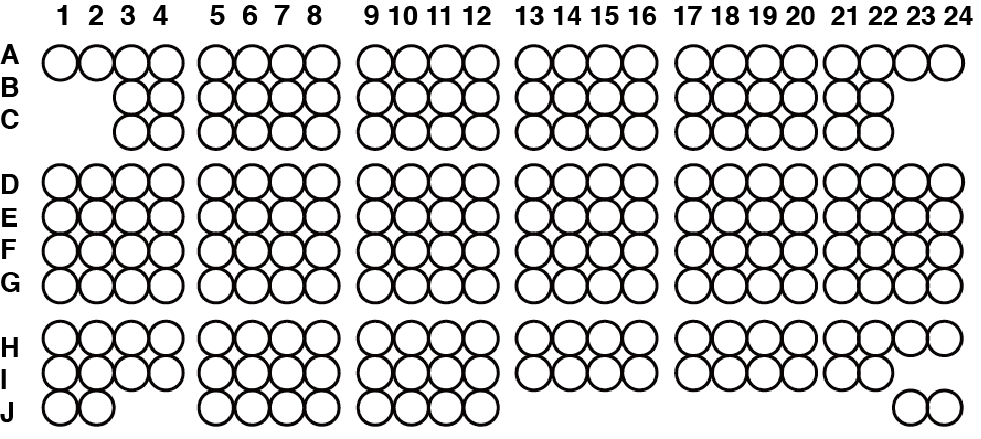 |
| --- | --- |
| Before immunotherapy (day 10) | 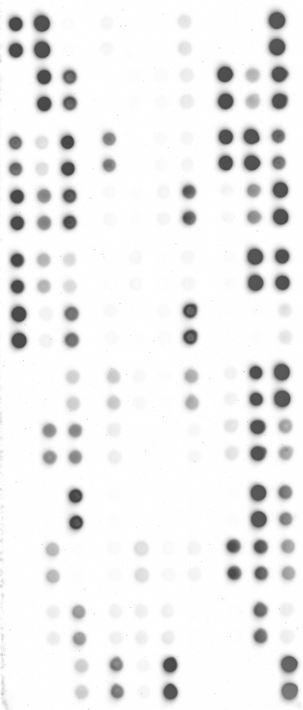 |
| During immunotherapy (day 28) | 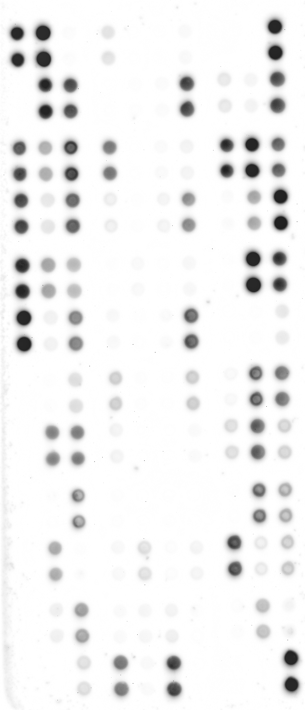 |
| After immunotherapy (day 42) | 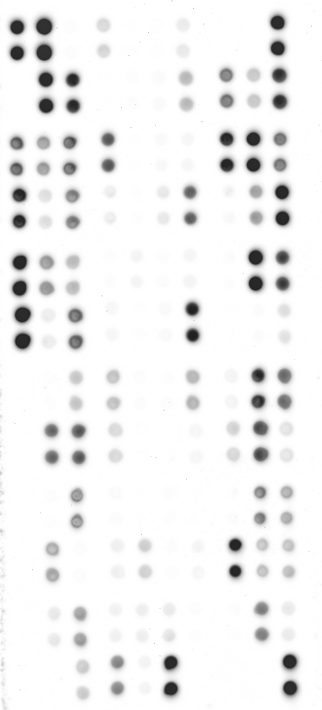 |

**Supplementary 3. Cytokine concentration determined by Luminex Assay**

| (pg/ml) | BAFF | sCD40L | TARC/CCL17 | C5/C5a | BDNF |
| --- | --- | --- | --- | --- | --- |
| day 10 | 453.3 ± 15.4 | 4647.2 ± 115.2 | 567.1 ± 27.6 | 8577.1 ± 342.2 | 10755.3 ± 140.6 |
| day 28 | 167.7 ± 9.7 | 1945.7 ± 48.9 | 286.0 ± 97.1 | 14554.5 ± 1056.3 | 10543.9 ± 261.3 |
| day 42 | 175.7 ± 4.6 | 3189.6 ± 144.5 | 286.0 ± 97.1 | 9263.0 ± 586.0 | 4915.8 ± 57.8 |
