# Supplementary material for: Cytokine changes during treatment of anti-Caspr2 encephalitis: a case report
Source: BMC Neurol. 2020 Aug 13;20:299. doi: 10.1186/s12883-020-01879-x (PMC7425608; doi:10.1186/s12883-020-01879-x)
Supplement: Supplementary file 2 — Additional file 2: Supplementary 4. CARE checklist [file 12883_2020_1879_MOESM2_ESM.pdf]

CARE case report guidelines

CARE Checklist of information to include when writing a case report

(cc) BY-NC-ND

Topic	Item	Checklist item description	Reported on Line
Title	1	The diagnosis or intervention of primary focus followed by the words "case report"	Title page
Key Words	2	2 to 5 key words that identify diagnoses or interventions in this case report, including "case report"	Key words
Abstract (no references)	3a	Introduction: What is unique about this case and what does it add to the scientific literature?	Abstract, paragraph 1
	3b	Main symptoms and/or important clinical findings	Abstract, paragraph 2
	3c	The main diagnoses, therapeutic interventions, and outcomes	Abstract, paragraph 2
	3d	Conclusion—What is the main "take-away" lesson(s) from this case?	Abstract, paragraph 4
Introduction	4	One or two paragraphs summarizing why this case is unique (**may include references**)	Background, paragraph 2
Patient Information	5a	De-identified patient specific information.	Case presentation, paragraph 1
	5b	Primary concerns and symptoms of the patient.	Case presentation, paragraph 1
	5c	Medical, family, and psycho-social history including relevant genetic information	Case presentation, paragraph 1
	5d	Relevant past interventions with outcomes	Case presentation, paragraph 1
Clinical Findings	6	Describe significant physical examination (PE) and important clinical findings.	Case presentation, paragraph 1
Timeline	7	Historical and current information from this episode of care organized as a timeline	Case presentation, paragraph 1
Diagnostic Assessment	8a	Diagnostic testing (such as PE, laboratory testing, imaging, surveys).	Case presentation, paragraph 2
	8b	Diagnostic challenges (such as access to testing, financial, or cultural)	N/A
	8c	Diagnosis (including other diagnoses considered)	Case presentation, paragraph 2,3
	8d	Prognosis (such as staging in oncology) where applicable	Case presentation, paragraph 4
Therapeutic Intervention	9a	Types of therapeutic intervention (such as pharmacologic, surgical, preventive, self-care)	Case presentation, paragraph 4
	9b	Administration of therapeutic intervention (such as dosage, strength, duration)	Case presentation, paragraph 4
	9c	Changes in therapeutic intervention (with rationale)	N/A
Follow-up and Outcomes	10a	Clinician and patient-assessed outcomes (if available)	Case presentation, paragraph 4
	10b	Important follow-up diagnostic and other test results	Figure 1
	10c	Intervention adherence and tolerability (How was this assessed?)	N/A
	10d	Adverse and unanticipated events	N/A
Discussion	11a	A scientific discussion of the strengths AND limitations associated with this case report	Discussion, paragraph 10
	11b	Discussion of the relevant medical literature **with references**.	Discussion, paragraph 8,9
	11c	The scientific rationale for any conclusions (including assessment of possible causes)	Discussion, paragraph 11
	11d	The primary "take-away" lessons of this case report (without references) in a one paragraph conclusion	Discussion, paragraph 11
Patient Perspective	12	The patient should share their perspective in one to two paragraphs on the treatment(s) they received	N/A
Informed Consent	13	Did the patient give informed consent? Please provide if requested	Yes ☒ No ☐
